# The Hospital World

**Published:** 1910-07-16

**Authors:** 


### The Hospital World.

### Personalia.

A few years ago the late Mr. Tom Jones was honorary surgeon to the Manchester Royal Infirmary. On the outbreak of the South African war he left England to take charge of the Welsh Hospital as surgeon-in-chief. Like many other brave men he fell a victim to enteric and died at Springfontein. His memory has not been forgotten at Manchester, and indeed is now to live on in a bronze medallion which is the work and the gift of Mr. John Cassidy, the sculptor. Owens College, Manchester, was the first, we believe, to possess Mr. Jones's portrait, of which the one in the Infirmary is a replica. It was unveiled by Sir William Cobbett, Chairman of the Board of Management, and connects the new infirmary with the workers of the earlier and parent institution, before the great hospital of to-day was more than thought of. Every hospital is the better for such artistic memorials as these.

The Royal Southern Hospital, Liverpool, has just held a complimentary dinner in honour of Dr. William Alexander, who, after twenty-two years' work as a surgeon, has resigned. Mr. William Adamson, the hospital's chairman, presided at the dinner, and, after the toast of "The Hospital" had been honoured, he presented Dr. Alexander with a portrait, the work of Mr. Frank T. Copnall, which had been subscribed for by the staff and committee of the Royal Southern. The best idea of Dr. Alexander's fine record, both as a surgeon and as a hospital worker, can be gained from the speech in which Mr. Robert Jones proposed his health. "As a surgeon," said Mr. Jones, "Dr. Alexander's many triumphs are to be found recorded in surgical literature. He came to Liverpool from Ireland a very distinguished student, and in Liverpool he took his primary fellowship and final fellowship of the R.C.S. in successive weeks. Dr. Alexander also took the Jacksonian prize in 1881, and in 1883 he gained the Astley Cooper prize, an achievement of which any surgeon might be proud. No surgeon before him had taken either of these prizes from Liverpool, and no surgeon since. It is not merely as a brilliant operator, but as a philosophic surgeon and pioneer, that Dr. Alexander has achieved distinction for himself and his hospital." In a witty and informing speech Dr. Alexander thanked the hospital for the honour it had paid to him, and remarked on the rapid development of surgery in his day.

At a garden party given to the staff of the St. Marylebone Infirmary by the Infirmary Committee on Wednesday last week, Dr. J. R. Lunn, F.R.C.S., who is shortly relinquishing his appointment as medical superintendent of the infirmary, was the recipient of a very gratifying presentation. Mr. John A. Merchant opened the proceedings on behalf of the Presentation Committee, and asked Dr. Elliott Browne, on behalf of the assembled staff and friends, to make the presentation, to which all present had contributed. Dr. Browne recalled the fact that twenty years ago that very day his late Majesty King Edward VII. and Queen Alexandra—then Prince and Princess of Wales—opened the infirmary, and of all the members then composing the committee and staff there remained only Dr. Lunn and Mr. Merchant. Dr. Lunn's services were not only appreciated by the Guardians, but also by the Local Government Board, who had expressed regret at his impending retirement. Dr. Lunn's had been a long and honourable career, and the note of regret they all felt was that ill-health should cause early retirement. In the name of all he expressed the hope of restoration to health of Dr. Lunn, and asked him to accept a chiming clock, which bore the following inscription: "Presented to Dr. J. R. Lunn, F.R.C.S. (Eng.), upon his retirement from the St. Marylebone Infirmary as medical superintendent, by the members of the staff (past and present), also a few friends. June 29, 1881—June 29, 1910." He was also asked to accept a solid silver inkstand and dish, inscribed similarly, as a token of their esteem.

### Elections and Appointments.

Mr. William Cordiner and Mr. George J. Walker have been appointed visitors to the Aberdeen Royal Infirmary for July.

The Rev. R. W. Thompson, the Rev. H. E. Steedman, Lieut.-Col. J. May, Mr. Jackson, Mr. Lefroy, Mr. Mares, and Mr. Tigwell have been elected to the Committee of the Basingstoke Cottage Hospital.

Ex-Provost Grant has been reappointed president of the Arbroath Infirmary, Forfar, and Mr. Francis Webster, Provost Alexander, and Mr. A. R. Duncan, Parkhill, vice-presidents. Mr. Charles Milne, Hillcrest, has been added to the directors.

At the Morningfield Hospital, Aberdeen, the following sub-committees have been appointed for the ensuing year: House and Finance—Mr. Maitland (convener), Sir John Fleming, Mr. Abernethy, Mr. Davidson, Mr. Glegg, Mr. Alexander Machray, Mr. Smith, and Mr. Walker; Committee on Applications for Admission—Colonel Youngson (convener), Mr. England, Mr. Grant, Mr. M'Donald, Mr. Maitland, Mr. Shepherd, and Mr. White; Ladies' Committee—Miss Georgina Allardyce, Miss Bremner, Mrs. Wardlaw Burnet, Mrs. Gray Campbell Fraser, Mrs. James Murray Garden, Mrs. Georges, Mrs. Alexander Ogston, Miss Thom, Mrs. James Walker, Mrs. Hall Wilson. Mr. Glegg, Mr. Maitland, and Mr. White have been appointed as a sub-committee to consider and report (1) whether it is desirable to accommodate, unless in exceptional circumstances, more patients than the annual revenue warrants; and (2) whether it would not be advisable to treat all legacies as capital and to carry the amounts of these to the endowment fund.

The Right Hon the Earl of Crawford, K.T., has been elected president of the Royal Albert Edward Infirmary, Wigan. The list of the new vice-presidents is as follows: Colonel Woods, Sir Francis S. Powell, Bart., Mr. J. C. Eckersley, Mr. Will Johnson, Mr. Edmund Littler Johnson, Mr. A. E. Johnson, Mr. Robert Thorley Johnson, M.A., Dr. M. Benson, Dr. R. Prosser White, Mr. Harold Sumner, Mr. A. M. Lamb, Lieut.-Colonel N. ffarington Eckersley, Mr. J. Stone, and Mr. J. Hodges (constituted life vice-presidents under Rule 56); the Mayor of Wigan for the time being, the ex-Mayor of Wigan for the time being, the Rector of Wigan (Rev. Canon R. G. Matthew), Lieut.-Colonel Sir W. E. M. Tomlinson, Bart., Lord Balcarres, M.P., Mr. Alfred Hewlett, Mr. C. E. Beazer, Mr. Henry L. W. Standish, Mr. H. J. Walmesley, Mr. H. S. Woodcock, Mr. Walter Mayhew, Colonel R. A. ffarington, Mr. T. Ratcliffe Ellis, Mr. James Marsden, Mr. Joe Coop, Mr. John Browne, Mr. William Berry, Mr. W. A. McClure, Mr. Jon. Phillips, Mr. W. D. Brown, Mr. T. M. Percy, Mr. James Brown, Mr. T. Brown, Mr. W. Mitchell Roocroft, Mr. R. B. Mawson, Mr. Chas. Appleton, Mr. Wm. Clark, Mr. Stephen Walsh, M.P., and Mr. H. Twist, M.P.

